# Effects of 8-Year Nitrogen and Phosphorus Treatments on the Ecophysiological Traits of Two Key Species on Tibetan Plateau

**DOI:** 10.3389/fpls.2018.01290

**Published:** 2018-09-11

**Authors:** Dan Wang, Tianqi Ling, Pengpeng Wang, Panpan Jing, Jiazhi Fan, Hao Wang, Yaoqi Zhang

**Affiliations:** ^1^International Center for Ecology, Meteorology and Environment, Jiangsu Key Laboratory of Agricultural Meteorology, Nanjing University of Information Science and Technology, Nanjing, China; ^2^School of Forestry and Wildlife Sciences, Auburn University, Auburn, AL, United States

**Keywords:** photosynthesis, stomatal conductance, intrinsic water-use efficiency, integrated water-use efficiency, stomatal slope parameter

## Abstract

Understanding how nitrogen (N) and/or phosphorus (P) addition affects plants carbon- and water- related ecophysiological characteristics is essential for predicting the global change impact on the alpine meadow ecosystem structure and function in carbon and water cycling. The Qinghai-Tibetan Plateau (QTP) with the largest alpine meadow in the world is regarded as the third pole in the earth and has been experiencing increased atmospheric N deposition. In this project, we focused on two key species (*Elymus dahuricus* and *Gentiana straminea*) of the alpine meadow on the Tibetan Plateau and investigated the variability of photosynthetic and stomatal responses to 8-year N and/or P treatments through field measurements and modeling. We measured photosynthesis- and g_s_-response curves to generate parameter estimates from individual leaves with two widely used stomatal models (the BWB model and MED model) for validation of growth and ecosystem models and to elucidate the physiological basis for observed differences in productivity and WUE. We assessed WUE by means of gas exchange measurements (WUE_i_) and stable carbon isotope composition (Δ^13^C) to get the intrinsic and integrated estimates of WUE of the two species. P and N+P treatments, but not N, improved the photosynthetic capacity (A_net_ and V_cmax_) for both species. Stomatal functions including instaneous measurements of stomatal conductance, intrinsic water-use efficiency and stomatal slope parameters of the two widely used stomatal models were altered by the addition of P or N+P treatment, but the impact varied across years and species. The inconsistent responses across species suggest that an understanding of photosynthetic, stomatal functions and water-use should be evaluated on species separately. WUE estimated by Δ^13^C values had a positive relationship with A_net_ and g_s_ and a negative relationship with WUE_i_. Our findings should be useful for understanding the underlying mechanisms of the response of alpine plants growth and alpine meadow ecosystem to global change.

## Introduction

Terrestrial ecosystems worldwide are limited or co-limited by nutrients, especially by nitrogen (N) and phosphorus (P) (Elser et al., 2007; LeBauer and Treseder, 2008; Harpole et al., 2011). While it is well-known that N and P addition typically increases plant growth, less is known about how N and P addition affects plants ecophysiological characteristics related to carbon and water acquisition. The Qinghai-Tibetan Plateau (QTP) is regarded as the third pole and has one of the largest alpine grasslands in the world. The QTP has been experiencing much greater than average global changes, such as increased atmospheric N deposition and climate warming (IPCC, 2013). The wet nitrogen deposition was estimated 6.96–7.55 kg N hm^-2^ y^-1^ on the QTP (Lv and Tian, 2007). With the increase of global nitrogen deposition and the relative slow mineralization rate due to the low temperature at the high elevation, it is critical to investigate the ecophysiological responses of the alpine grasslands species to N and P addition. The information will be valuable in predicting the global change impact on the alpine meadow ecosystem structure and function in carbon and water cycling.

Alpine meadow system on the Tibetan Plateau is characterized as low N and P availability due to the slow mineralization processes at the low temperature. The addition of N and P is therefore anticipated to boost the growth of the alpine meadow species. Yang et al. (2014) reported that N and P additions both increased the aboveground biomass on QTP alpine meadow and the P effect was more evident than the N effect. Fu and Shen (2017) synthesized 51 studies on the QTP and confirmed that nitrogen addition significantly increased plant height and aboveground biomass. Photosynthetic carbon gain of leaves was mainly affected by N concentration and light availability (Field and Mooney, 1986). This observation is supported by the positive relationships between leaf N concentration and net photosynthesis observed in many different species (Turnbull et al., 2007; Wang et al., 2012). However, whether the alpine meadow species is photosynthetically N or P limited and whether different species respond to N/P addition differently remains unknown. Chlorophyll fluorescence parameters, stomatal conductance (g_s_) and maximum rate of carboxylation (V_cmax_) are important physiological parameters related to plant photosynthesis. All these physiological parameters are nutrient-dependent and probably affected under N and/or P addition conditions (Reich et al., 2009; Liu and Greaver, 2010). Measurements of photosynthetic and stomatal responses of the alpine meadow species to N and/or P addition are needed for validation of plant growth models and to elucidate the physiological basis for observed differences in plant growth responses to the addition of N and/or P.

Successfully simulating canopy and ecosystem photosynthesis and transpiration requires understanding the rate-impacting factors in leaf photosynthesis and stomatal activities (Laisk et al., 2005). Understanding and predicting larger scale carbon, water, and energy cycles also requires accurate estimates of the leaf diffusive (stomatal) conductance to water vapor using stomatal conductance models. The regulatory role of stomata in photosynthetic CO_2_ assimilation and water vapor loss to the atmosphere is arguably the most fundamental constraint on plant function and most critical process in simulating and predicting larger scale carbon, water and energy fluxes. Empirical and mechanical models have been incorporated into land surface models to simulate stomatal conductance. The Ball, Woodrow & Berry (BWB model) and Medlyn model (MED model) are two widely used stomatal models to describe the complex behavior of stomata at the leaf level (Medlyn et al., 2001; Wolz et al., 2017). The parameters of these models (m and g_0_ from BB model, g_1_ and g_0_ from MED model) are valuable for large-scale simulations and represent important physiological traits that determine plant water-use efficiency. Compared with instant measurements, the changes in stomatal slope parameters (m and g1) with plant’s biophysical environment provide a simple but synthetic framework for studying climate-change related carbon and water cycling, because of its sensitivity to CO_2_, vapor pressure deficient, and photosynthesis, as well as its crucial information about climate change impacts on photosynthesis and water-use efficiency (Oren et al., 1999). How stomatal slope parameters of alpine meadow species varies among different species and at different fertilization conditions requires further study and analysis.

Through gas exchange measurements, WUE can be expressed as intrinsic WUE (WUE_i_, the ratio of net photosynthesis to stomatal conductance, A_net_/g_s_). Integrative WUE (Δ^13^C) can be assessed indirectly with measurements of the stable carbon isotope composition (δ^13^C) of leaves or other plant materials. This latter method is based on the linear relationship between δ^13^C and the ratio of the concentration of CO_2_ inside and outside of the leaf (Farquhar et al., 1982). Using gas exchange measurements (WUE_i_) and carbon isotope composition would provide both instantaneous and integrated estimates of WUE (Seibt et al., 2008). Whether the integrated measurements matches with the instantaneous measurements of water-use efficiency, stomatal slope, and photosynthetic parameters for the alpine meadow species requires further investigation and analysis.

Previous studies indicated that N and P additions increased the aboveground biomass of grass but decreased forb biomass (Yang et al., 2014; Fu and Shen, 2017). To identify the ecophysiological responses of different PFTs to N/P addition, we will select two key species (*Elymus dahuricus*, a C_3_ perennial grass and *Gentiana straminea*, a C_3_ perennial forb) of the alpine meadow on the Tibetan Plateau and investigate the variability of photosynthetic and stomatal responses to N or P additions and associated leaf traits through field measurements and modeling. We measured photosynthesis- and g_s_-response curves to generate parameter estimates from individual leaves for two widely used stomatal models (the BWB model and MED model). We assessed WUE by means of gas exchange measurements (WUE_i_) and stable carbon isotope composition (Δ^13^C) to get the intrinsic and integrated estimates of WUE of the two key alpine meadow species. The objectives of this study were (1) to determine whether P or N or both was the nutrient more limiting to the photosynthesis of two alpine meadow species growing in the field; (2) to investigate whether long-term fertilization treatments changes the stomatal slope parameters; (3) to identify the relationship of leaf traits to integrated water-use efficiency (Δ^13^C). We hypothesized that (1) as the effect on the aboveground biomass, both P and N addition will improve the photosynthetic capacity of the two species and the P effect will be more evident than the N effect; (2) the addition of N or P will not change the stomatal regulating properties; (3) the integrated water-use efficiency (Δ^13^C) will be correlated with the instantaneous measurements of water-use efficiency (WUE_i_ and stomatal slope parameters).

## Materials and Methods

### Site Description

The study site was established in an alpine grassland at the Haibei Alpine Meadow Ecosystem Research Station (37°37^′^ N, 101°12^′^ E, 3240 m above the sea level), located on the northeastern Tibetan Plateau in Qinghai Province, China (Yang et al., 2014; Song and Yu, 2015). The historic mean annual temperature is -1.7°C and annual precipitation is 560 mm, 85% of which occurs in the growing season from May to September. The PAR (photosynthetically active radiation) reaches 370 W m^-2^ s^-1^ in the growing season, equivalent ot 10 MJ m^-2^ d^-1^. The mean annual temperature was 2.89 and -0.02°C and the mean annual precipitation was 601 and 453 mm in 2015 and 2016, respectively. The mean daily day- and night-temperature and maximal temperature was 11.6, 4.6, and 28.4°C and 13.6, 5.2, and 28.9°C in the growing season in 2015 and 2016, respectively. The soil is classified as Mat Cry-gelic Cambisols (Chinese Soil Taxonomy Research Group, 1995), corresponding to Gelic Cambisol. Topsoil (0–10 cm) has a pH value of 7.5, and contains 71.4 g kg^-1^ organic C, 7.8 g kg^-1^ total N, and 0.77 g kg^-1^ total P before nutrient treatments were applied in 2009 (Huang et al., 2014). The experimental site was fenced before the experiment plot was established. The plant community at the experimental site is dominated by *Kobresia humilis, Stipa aliena, Elymus nutans, E. dahuricus, G. straminea*, and *Festuca ovina*.

### Experimental Design and Sampling

The experimental design followed the standard protocols of Nutrient Network (NutNet^1^). In mid-May 2009, an experimental area of 1 ha was fenced to prevent grazing disturbance. Twenty-four plots of 6 m × 6 m were randomly assigned to four treatments with six replicates (blocks) in a complete randomized block design. The blocks were separated by a 2-m-wide buffer zone, and the plots within each block were separated by a 1-m-wide buffer zone to minimize disturbance from neighboring treatments. The four treatments consisted of the following: (1) Control (CK, no fertilizer was added); (2) N addition (in the form of urea, 100 kg N ha^-1^ year^-1^); (3) P addition (in the form of triple superphosphate, 50 kg ha^-1^ year^-1^); and (4) N+P addition (combined addition of N and P in the same amounts as the solo treatments). Pelletized fertilizer was evenly distributed by hand onto the plots after sunset in July from 2009 to 2016.

### Photosynthetic Measurements

Gas exchange (including net photosynthetic rate and stomatal conductance) was measured with a portable infrared gas analyzer (LI-COR 6400LCF; LI-COR, Lincoln, NE, United States) on 1 randomly selected fully expanded healthy leaf from each plot of each treatment in August, 2015 and 2016. During measurements, leaves were exposed to a CO_2_ concentration of 370 μmol mol^-1^, leaf temperature of 25°C, and airflow through the chamber of 300 μmol s^-1^. Leaves were acclimated to a photosynthetic photon flux (PPFD; 2000 μmol m^-2^ s^-1^) until photosynthetic rates stabilized. The rate of photosynthesis at a PPFD of 2000 μmol m^-2^ s^-1^ was defined as the net photosynthetic rate (A_net_). PSII efficiency in light-adapted leaves (The stomatal functions including instantaneous measurements of stomatal conductance) and PSII operating efficiency (Φ_PSII_) were also measured using a Licor 6400-40 Leaf Chamber Fluorometer. The photosynthesis-CO_2_ response (A–*C*_i_) curves were measured each year in the middle of the growing season (August). During measurement, leaves were acclimated for 30–60 min before adjusting the CO_2_ concentrations. Thereafter, CO_2_ concentration was decreased in five steps (400, 300, 200, 100, and 50 ppm CO_2_) and then increased in four steps (400, 600, 800, and 1000 μmol mol^-1^ CO_2_). A–C_i_ curves were fit to the Farquhar-von Caemmerer-Berry model based on the methods developed by Miao et al. (2009). By using grid search and non-linear two-stage least square regression technique, the fitting model solves the A–C_i_ parameters including maximum ribulose 1⋅5-bisphosphate carboxylase/oxygenase (Rubisco) carboxylation rate (V_cmax_, μmol m^-2^ s^-1^) and potential light saturated electron transport rate (J_max_, μmol m^-2^ s^-1^), respectively.

Immediately following gas-exchange measurements, leaf samples were oven-dried till constant weight. Leaf samples were then ground and N concentration (N_mass_, mass based nitrogen concentration) were measured with a Perkin Elmer CHN Analyzer (Model 2400).

### Integrated Water-Use Efficiency (Δ^13^C)

Leaves were oven-dried at 65°C for 2 weeks, then ground to fine powder. Approximately, 2 mg of homogenized leaves were weighed into tin capsules and analyzed with an elemental analyzer coupled to an isotope ratio mass spectrometer (Elemental combustion system 4010, Costech instruments). Carbon isotope ratios were expressed in conventional δ notation and referenced to the Pee Dee Belemnite (PDB) standard for δ^13^C. Measurement error was less than 0.3‰ for δ^13^C. The carbon isotope composition (δ^13^C) was calculated as the ratio (‰):

$${\text{δ}}^{13}C=\left[\left(\frac{R_{\text{sample}}}{R_{\text{standard}}}\right)-1\right]\times 1000$$

Carbon isotope ratio values were converted to discrimination values (Δ, ‰) by the equation (Farquhar et al., 1989):

$$\Delta =(\text{δa}-\text{δp)/}\left(1+\frac{\text{δp}}{1000}\right)$$

Where δa is the carbon isotope ratio of CO_2_ in the atmosphere (assumed to be -8 pars per mil, Seibt et al., 2008) and δp is the measured carbon isotope ratio of the leaf tissue. Lower values of Δ indicate higher water-use efficiency values.

### Stomatal Slope Parameter Calculations

The Ball et al. (1987) (Eq. 1) or Medlyn et al. (2001) (Eq. 2) models of *g_s_* were used to calculate the stomatal slope parameters (m and g1).

$$Eq.\;1\;g_{s}=g_{0}+m\frac{Ah}{C_{a}}$$

where *g_s_* is stomatal conductance (mol m^-2^ s^-1^), *A* is the net rate of photosynthetic CO_2_ uptake (μmol m^-2^ s^-1^), *h* is atmospheric relative humidity (unitless), *C_a_* is the atmospheric CO_2_ concentration at the leaf surface (μmol mol^-1^), *g_0_* is the y-axis intercept and *m* is the slope of the line.

$$Eq.\;2\;g_{s}=g_{0}+1.6\left(1+\frac{g_{1}}{\sqrt{D}}\right)\frac{A}{C_{a}}$$

where *D* is atmospheric vapor pressure deficit (kPa) and *g_1_* is the model parameter related to the slope of the line.

For each leaf, a linear least squares regression of Eq. 1 or Eq. 2 was used to estimate the intercept and slope parameters of the Ball et al. (1987) (*3*) model and Medlyn et al. (*5*) model, respectively. Biologically, the slope parameter of each model represents the sensitivity of *g*_s_ to changes in A_net_, C_a_ and atmospheric water status and will be the focus of this analysis. A term for the *y* intercept of each model algorithm (*g*_0_) can be used to describe variation in minimum *g*_s_. Only leaves that provided a regression between modeled and observed stomatal conductance with an R^2^ > 0.8 were included in further analyses (Wolz et al., 2017).

### Statistical Analysis

Three-way analysis of variance (ANOVA) was used to test the fixed effects of year, species, fertilization treatment and their interaction on A_net_, g_s_, WUE_i_, N_mass_, ${\text{F}}_{\text{v}}^{\prime }/{\text{F}}_{\text{m}}^{\prime }$, Φ_PSII_, V_cmax_, J_max_, and Δ^13^C. *Post hoc* Tukey HSD tests were conducted on specific contrasts to examine significant treatment effects among groups. General linear models (GLMs) were used to assess the relationship between Δ^13^C, WUE_i_ and other physiological parameters. For all tests, the normality of the residuals was tested using the Shapiro–Wilk test. All statistical testes were considered significant at *p* ≤ 0.05. Mean values of each variable were expressed with their standard error (SE). All analyses were conducted in R (R 2.14^2^).

## Results

**FIGURE 1:**
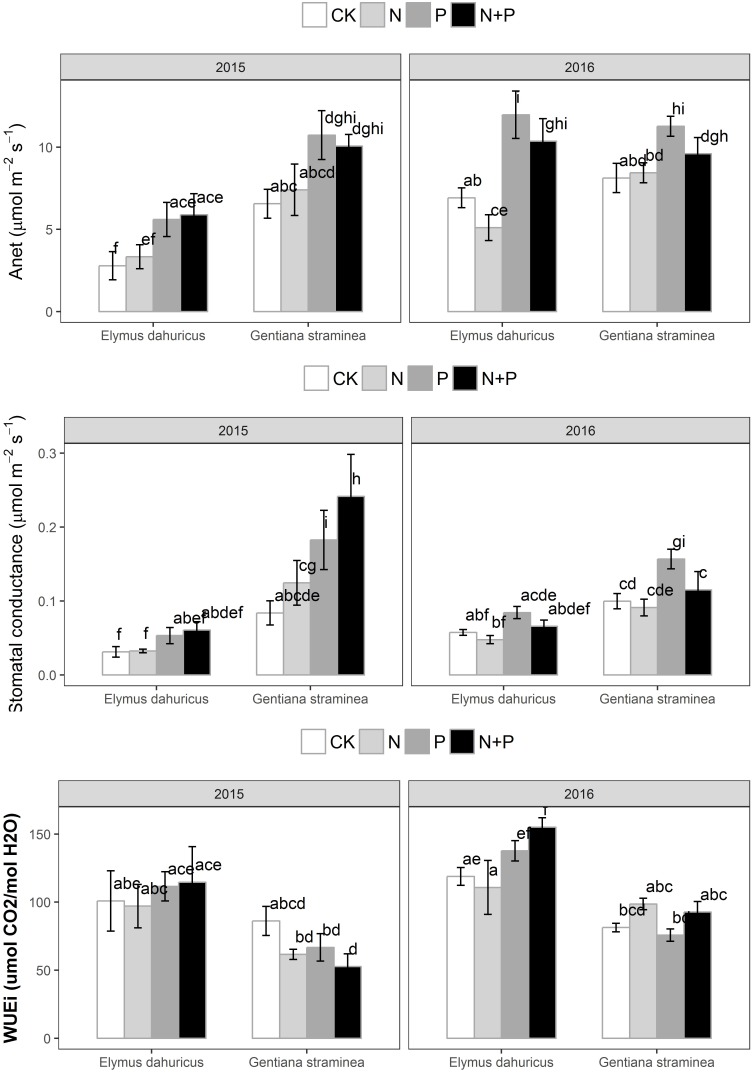
The response of net photosynthetic CO_2_ assimilation (A_net_), stomatal conductance (_*g*s_), and intrinsic water-use efficiency (WUE_i_) to the nutrient addition for *Elymus dahuricus* and *Gentiana straminea*. Measurements were taken at CK (control), N (nitrogen), P (phosphorus), and N+P (nitrogen and phosphorus) treatments in the peak growing season in 2015 and 2016. Values are means ± 1 SE; *n* = 5. Bars sharing the same letters are not significantly different.

The three-way ANOVA analysis revealed that effects of nutrient additions on photosynthetic traits varied among species, years, treatment and their interactions (**Figure 1** and **Table 1**). Photosynthetic and leaf traits varied between years and among species, with *G. straminea* possessing higher A_net_, g_s_, ${\text{F}}_{\text{v}}^{\prime }/{\text{F}}_{\text{m}}^{\prime }$, Φ_PSII_, V_cmax_, J_max_, N_mass_, and Δ^13^C and lower WUE_i_ and stomatal slope parameters compared with *E. dahuricus* (**Table 1**). A_net_ of plants with P and N+P treatments was significantly higher than those with N and CK treatments for *E. dahuricus* and *G. straminea* in 2015 and 2016 (**Figure 1**). Across species and years, the value of g_s_ of plants with P and N+P treatment was significantly higher than those with N and CK treatments. There were significant species, year and species ^∗^ treatment effect on WUE_i_. The value of WUE_i_ of *E. dahuricus* with N+P and P treatment was significantly higher than those with N and CK treatments.

**Table 1:** Analysis of variance (*F*-value) of the effects of species, treatment, year and their interactions on net photosynthetic rate (A_net_), stomatal conductance (g_s_), intrinsic WUE (WUEi), leaf nitrogen concentration (N_mass_), the maximal PSII efficiency in the light (${\text{F}}_{\text{v}}^{\prime }/{\text{F}}_{\text{m}}^{\prime }$), the actual PSII efficiency (Φ_PSII_), maximal carboxylation rate (V_cmax_), potential light saturated electron transport rate (J_max_), stomatal slope parameters m and g1, and Δ^13^C.

Variation	A_net_	g_s_	*iWUE*	${\text{F}}_{\text{v}}^{\prime }/{\text{F}}_{\text{m}}^{\prime }$	Φ_PSII_	V_cmax_	J_max_	g1	m	N_mass_	Δ^13^C
Species	8.9**	96.9***	48.7***	110.4***	79.3***	4.9*	4.1*	9.9***	12.3***	565.6***	264.7***
Treatment	19.8***	11.5***	1.6	17.8***	11.6***	7.5***	8.2**	1.0	0.8	10.7***	2.9*
Year	19.9***	0.8	12.2***	48.9***	61.2***	21.9***	14.9***	15.4***	27.3***	15.8***	0.1
Species × treatment	1.1	3.3*	3.2*	4.6**	2.0	2.8*	0.7	0.8	0.3	0.4	0.3
Species × year	6.9**	15.1***	0.0	1.4	4.5*	5.2*	6.8**	2.7	0.7	8.0**	167.7***
Treatment × year	0.7	3.6*	0.9	1.7	1.0	1.8	2.6*	4.1**	4.1**	1.5	0.4

*^∗∗∗^ Indicates significance level at 0.001, ^∗∗^ 0.01, ^∗^ 0.05*.

**FIGURE 2:**
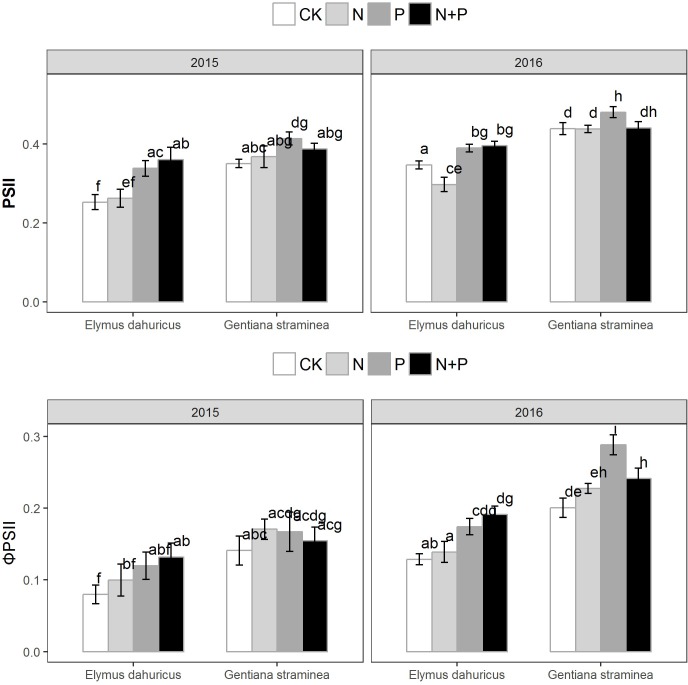
The response of the maximal PSII efficiency in the light (${\text{F}}_{\text{v}}^{\prime }/{\text{F}}_{\text{m}}^{\prime }$) and the actual PSII efficiency (Φ_PSII_) to the nutrient addition for *E. dahuricus* and *G. straminea*. Measurements were taken at CK (control), N (nitrogen), P (phosphorus), and N+P (nitrogen and phosphorus) treatments in the peak growing season in 2015 and 2016. Values are means ± 1 SE; *n* = 5. Bars sharing the same letters are not significantly different.

Significant effects were detected among species, year, treatment and species × treatment for ${\text{F}}_{\text{v}}^{\prime }/{\text{F}}_{\text{m}}^{\prime }$ and Φ_PSII_ (**Figure 2** and **Table 1**). For *E. dahuricus*, plants with P and N+P treatments had higher ${\text{F}}_{\text{v}}^{\prime }/{\text{F}}_{\text{m}}^{\prime }$ and Φ_PSII_ than those with CK and N treatments. For *G. straminea*, P addition significantly increased ${\text{F}}_{\text{v}}^{\prime }/{\text{F}}_{\text{m}}^{\prime }$ in 2016 and 2017 compared with CK treatments and Φ_PSII_ compared with CK, N and N+P treatments in 2017.

**FIGURE 3:**
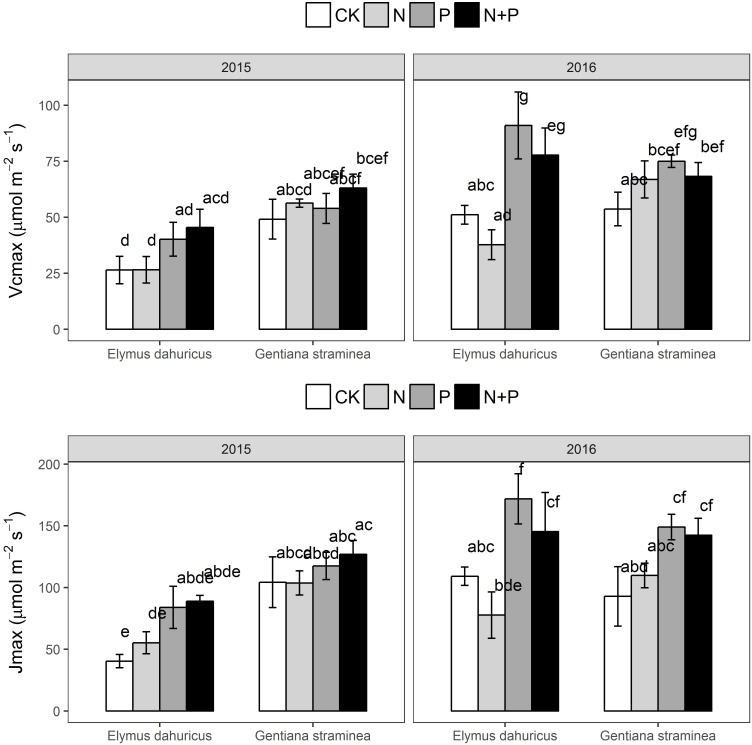
The response of maximum rate of Rubisco activity (V_cmax_) and potential light saturated electron transport rate (J_max_) to the nutrient addition for *E. dahuricus* and *G. straminea*. Measurements were taken at CK (control), N (nitrogen), P (phosphorus), and N+P (nitrogen and phosphorus) treatments in the peak growing season in 2015 and 2016. Values are means ± 1 SE; *n* = 5. Bars sharing the same letters are not significantly different.

There were significant effects of species, year, treatment and their interactions for V_cmax_ and J_max_ (**Figure 3** and **Table 1**). Across *E. dahuricus* and *G. straminea* in 2 years, plants with P and N+P treatments had higher V_cmax_ and J_max_ than those with N and CK treatments.

**FIGURE 4:**
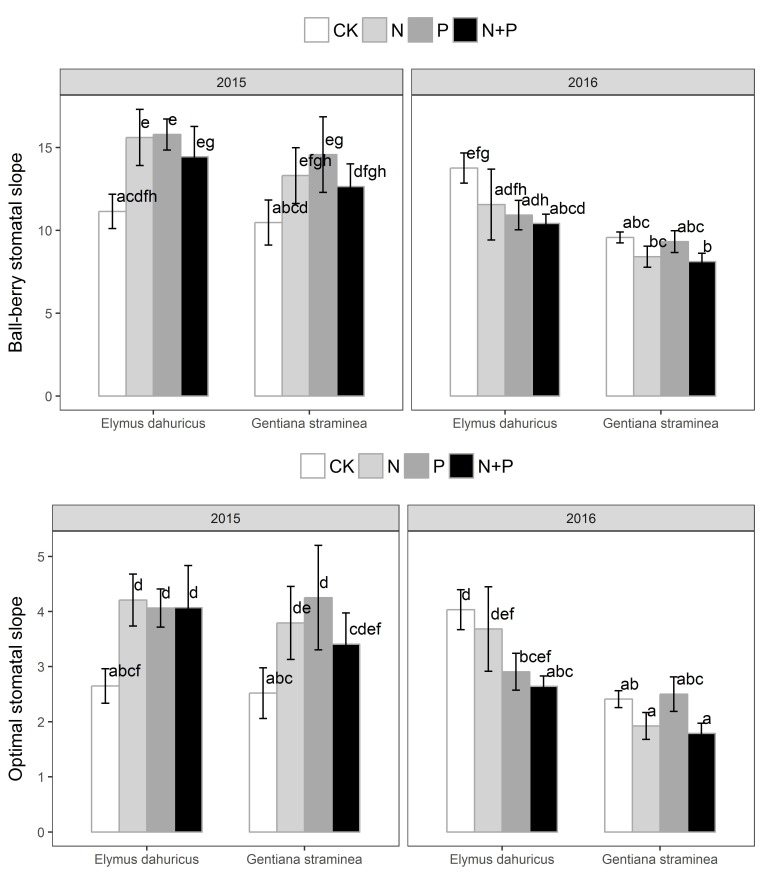
The response of stomatal slope m from Ball-berry model and g1 from Medlyn model to the nutrient addition for *E. dahuricus* and *G. straminea*. Measurements were taken at CK (control), N (nitrogen), P (phosphorus), and N+P (nitrogen and phosphorus) treatments in the peak growing season in 2015 and 2016. Values are means ± 1 SE; *n* = 5. Bars sharing the same letters are not significantly different.

There were no treatment, but species, year and treatment × year effects on stomatal slope parameters of m and g1 (**Figure 4** and **Table 1**). Variation in estimates of the g1 slope parameter from the Medlyn et al. model mirrored that of m, both in species rank and treatment effects. In 2015, plants with nutrient treatment had higher values of m and g1 than plants with CK treatments for both species. In 2016, plants with P and N+P treatments had lower values of m and g1 than plants with CK treatment for *E. dahuricus*. There were no significant treatment effects for *G. straminea* in 2016.

**FIGURE 5:**
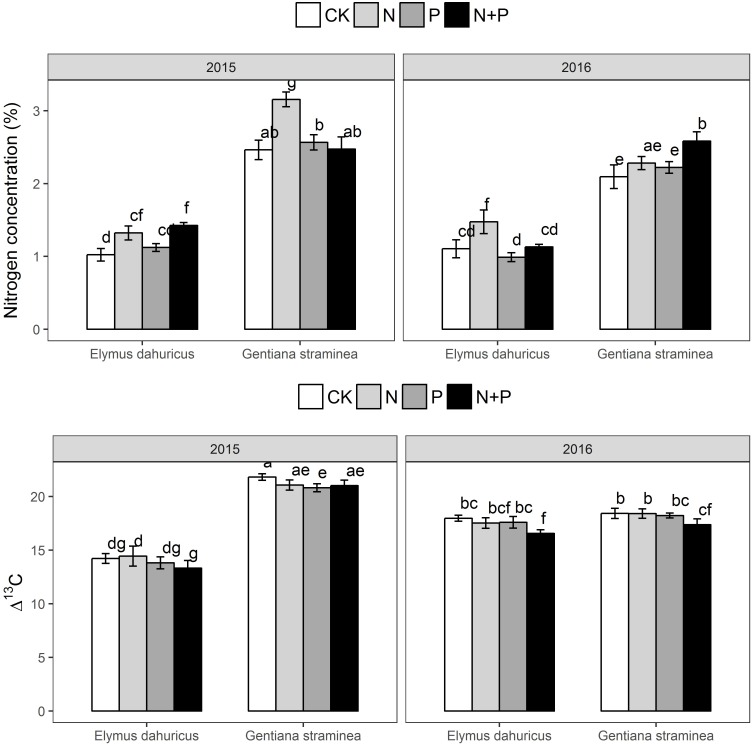
The response of leaf N (N_mass_) and integrated water-use efficiency (Δ^13^C) to the nutrient addition for *E. dahuricus* and *G. straminea*. Measurements were taken at CK (control), N (nitrogen), P (phosphorus), and N+P (nitrogen and phosphorus) treatments in the peak growing season in 2015 and 2016. Values are means ± 1 SE; *n* = 5. Bars sharing the same letters are not significantly different.

Significant effects were detected among species, years, treatment and their interactions for N_mass_ and Δ^13^C (**Figure 5** and **Table 1**). Across the two species in the 2 years, plants with N and N+P treatment had higher N_mass_ than plants with P and CK treatments. The value of Δ^13^C varied among species and treatments. Plants with N+P treatment had lower Δ^13^C than plants with CK treatments across two species.

**FIGURE 6:**
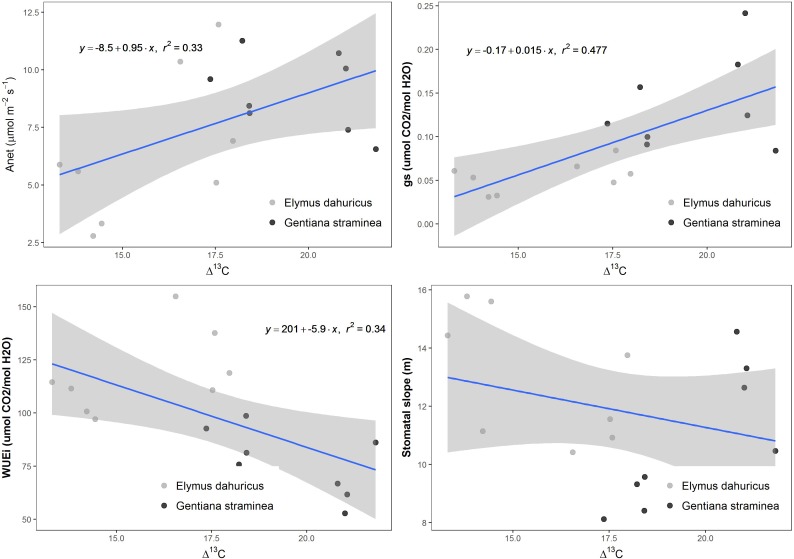
The linear relationship between integrated water-use efficiency (Δ^13^C) and net photosynthetic CO_2_ assimilation (A_net_), stomatal conductance (g_s_), intrinsic water-use efficiency (WUE_i_), stomatal slope m from Ball-berry for *E. dahuricus* and *G. straminea*. Only significant relationships (*p* < 0.05) with equations were shown on the figures.

Δ^13^C had a positive relationship with A_net_ and g_s_, a negative relationship with WUE_i_ and no relationship with stomatal slope parameter of m or g1 (**Figure 6**).

## Discussion

In order to investigate the variation in the ecophysiological responses to N and/or P treatment and detect whether there is stomatal acclimation with long-term fertilization treatment, we assessed leaf traits of two key species on Tibet alpine grassland across two growing seasons. The systematic measurements of gas exchange after long-term fertilization treatment are essential for validation of plant growth and ecosystem models and to elucidate the physiological basis for observed differences in the response of growth and water-use to N and/or P additions.

In our study, photosynthetic and leaf traits varied among species and treatment, with *G. straminea* possessing higher A_net_, g_s_, ${\text{F}}_{\text{v}}^{\prime }/{\text{F}}_{\text{m}}^{\prime }$, Φ_PSII_, V_cmax_, J_max_, N_mass_, and Δ^13^C and lower WUE_i_ and stomatal slope parameters compared with *E. dahuricus* (**Table 1**). Contrary to our first hypothesis, 8 years of N treatments had no effects on photosynthetic traits of either species, despite significant increases in foliar N for both species. No stimulation of photosynthetic traits by N treatment in the present study was not in line with other results from this experiment site demonstrating that N addition increased the aboveground biomass by 24% (Yang et al., 2014). Though nitrogen addition significantly increased plant productivity in the alpine meadow (Bassin et al., 2012; Fu and Shen, 2017) and other grasslands (LeBauer and Treseder, 2008; Lee et al., 2010), the effect of nitrogen addition on plants’ photosynthetic performance on alpine meadow has not been investigated widely. The results implied that the elevated foliar N might not have been partitioned to photosynthetic components, i.e., RuBP carboxylase (Rubisco) or chlorophylls (Bauer et al., 2004), suggesting a decoupling of photosynthesis and elevated foliar N. The proportion of N allocation to Rubisco may not increase, as shown by the maximum Rubisco carboxylation efficiency, which was not altered by N fertilization. The effect of N on plant growth is generally due to both an effect on photosynthesis and leaf growth, which was mostly confirmed on C_3_ species. Such long and high N treatments might have eliminated any N limitation to photosynthetic performances.

In consistent with earlier findings that P addition increased the aboveground biomass by 52% (Yang et al., 2014), P addition increased A_net_ for both species compared with CK treatment (**Figure 1**). The increase of A_net_ promoted by P addition may be attributed to increases in g_s_, ${\text{F}}_{\text{v}}^{\prime }/{\text{F}}_{\text{m}}^{\prime }$, Φ_PSII_ and Rubisco activity (V_cmax_ and J_max_) for both species (**Figures 1**, **3**). Phosphorus (P) nutrient is essential to a variety of plant functions and a major component of nucleic acids, sugar phosphates, ATP, and phospholipids, all of which play important roles in photosynthesis. Low leaf P is thought to limit A_net_ through reductions in ribulose-1,5-bisphosphate (RuBP) regeneration, carboxylation activity, light use efficiency, and stomatal conductance (Campbell and Sage, 2006; Thomas et al., 2006). It had been shown that P supply influenced partitioning of N to Rubisco and important for RuBP regeneration, suggesting there might be interactive effects of N and P availability on A_net_ (Warren et al., 2005). In a cross-biome analysis of the influence of P on the linear relationship between photosynthetic capacity (A_max_) and foliar N, the slope of such linear relationship increased with leaf P (Reich et al., 2009). The results in this study indicated there was no additive effect of N+P treatment on the photosynthetic capacity of these two species. In alpine ecosystems on the Tibetan Plateau, these two species were limited by P rather than N availability photosynthetically and P addition will trigger a stronger positive response of plant photosynthesis than N addition. The findings here suggest that it is important to learn more about the physiology of P effects on A_net_ for modeling carbon and biogeochemical fluxes and vegetation–climate interactions, especially for regions where low P supply may play a role in limiting plant and ecosystem function. The significant year ^∗^ species and year ^∗^ treatment effects also suggested that the meteorological conditions might also play a significant effect on the ecophysiological responses of the two key species to the nutrient treatments.

Nutrient addition not only affected plants carbon gain of these two species, but also their stomatal functions in water relations. P addition significantly increased g_s_ for *E. dahuricus* and *G. straminea*. P stimulation on stomatal conductance indicated that nutrient availability may limit stomatal function and thus was important for maximizing carbon gain. Higher stomatal conductance and thus higher transpiration can enhance nutrient uptake. Variation across PFTs and environmental gradients in the g_1_ and m parameters had been reported widely (Medlyn et al., 2011; Lin et al., 2015; Wolz et al., 2017). Consistent with our prediction, there was no altered stomatal sensitivity under different nutrient addition treatments. N, P or N+P treatment increased m and g1 in 2015 (*p* = 0.06 for *E. dahuricus* and for *G. straminea*). The slope parameter g1 and m (dimensionless) relating g_s_ to AH/cs was obtained by fitting the equation to leaf gas-exchange data (Leakey et al., 2006). The values of g1 and m are largely representative of the ratio g_s_/A, the reciprocal of intrinsic water-use efficiency (Franks et al., 2017). Therefore, it might be expected that plants with characteristically higher WUE_i_ will exhibit lower g1, which was the case for the stomatal slope parameter g1 and m in 2015 and 2016 for both species. The case study of 15 temperate C_3_ tree species revealed that long-held assumptions about stomatal function were a substantial source of error in physiological models of carbon and water fluxes at the leaf scale (Wolz et al., 2017). Current modeling approaches assuming a universal stomatal slope parameter under different conditions could probably propagate the errors to simulations of crop performance, ecosystem function and global biogeochemical cycles.

The ratio C_i_/C_a_, measured under normal (light-saturated) conditions of leaf gas exchange or as a time-integrated value from carbon isotope discrimination (Δ^13^C) in plant material, has long been recognized as an index of plant water-use efficiency. A decline in C_i_/C_a_ [and Δ^13^C] is equivalent to an increase in intrinsic water-use efficiency (Farquhar et al., 1989). This relationship can be difficult to resolve because the two variables integrate plant response over different time spans: WUE*_i_* is an instantaneous measurement while Δ^13^C is integrated over the growing season. There was evidence for a negative linear relationship between WUE_i_ and Δ^13^C in different species and functional types (Roussel et al., 2009; Orchard et al., 2010; Wang et al., 2012). Recent analyses had suggested that using Δ^13^C as an indicator of variation in WUE could be less effective when applied across species (Warren and Adams, 2006; Seibt et al., 2008; Wang et al., 2012; Cernusak et al., 2016). Our study showed that the diversity of Δ^13^C had a negative relationship with WUE_i_ of the two species, consistent with previous studies (**Figure 6**). Plants can increase WUE by increasing the efficiency of carbon fixation inside the leaf, either by increasing the efficiency of light harvesting or carboxylation processes. However, Δ^13^C was positively correlated with A_net_, implying that the decrease of WUE (increase of Δ^13^C) was more driven by the increase of g_s_. The overall Δ^13^C during carbon assimilation is dependent on the CO_2_ concentration at the sites of carboxylation, which in turn is strongly dependent on mesophyll conductance (g_m_). Many studies reported no significant relationship between Δ^13^C and WUE_i_ (Seibt et al., 2008; McCarthy et al., 2011), claiming mesophyll conductance contributed to the observed variability of Δ^13^C.

## Conclusion

This study provided an ecophysiological investigation of two alpine meadow species after 8-year N and/or P treatments with a systematic measurement of leaf traits across two growing seasons. P and N+P addition improved the photosynthetic capacity for both species. A_net_ of the two alpine species in this study responded similarly to N and/or P treatment and the P stimulation on the A_net_ was associated with increased g_s_, ${\text{F}}_{\text{v}}^{\prime }/{\text{F}}_{\text{m}}^{\prime }$ and V_cmax_ for *E. dahuricus* and *G. straminea*. The stomatal functions including instantaneous measurements of stomatal conductance, intrinsic water-use efficiency and the stomatal slope parameters of the two widely used stomatal models were altered by the addition of P or N+P treatment, but the impact varied across years or species. This suggests that an understanding of photosynthesis, stomatal functions, and water-use should be evaluated on species basis. The effectiveness of integrating Δ^13^C and intrinsic water-use efficiency was confirmed. Our findings should be useful for understanding the underlying mechanisms of the response of alpine plants to global change.

## Author Contributions

DW came up with the idea and manage the experimental sites and wrote the paper. TL, PW, PJ, HW, and JF conducted the experiment and analyzed the data. YZ helped with the manuscript writing.

## Conflict of Interest Statement

The authors declare that the research was conducted in the absence of any commercial or financial relationships that could be construed as a potential conflict of interest. The reviewer AG and handling Editor declared their shared affiliation.

## Footnotes

1 http://nutnet.umn.edu

2 http://www.r-project.org/

## Abbreviations

A_net_: net CO_2_ assimilation rate (umol m^-2^ s^-1^)
${\text{F}}_{\text{v}}^{\prime }/{\text{F}}_{\text{m}}^{\prime }$: the maximal photosystem II (PSII) efficiency in the light
g_s_: stomatal conductance (mol m^-2^ s^-1^)
J_max_: maximum electron transport rate (umol m^-2^ s^-1^)
N_mass_: leaf mass-based nitrogen concentration (%)
PFTs: plant functional types
Φ_PSII_: the actual PSII efficiency
RuBP: ribulose-1,5-bisphosphate carboxylase
V_cmax_: maximum carboxylation rate (umol m^-2^ s^-1^)
WUEi: intrinsic water-use efficiency (umol CO_2_/mol H_2_O)
Δ^13^C: stable isotope ^13^C discrimination values (‰).
